# Core Decompression Combined with Intraosseous Autologous Conditioned Plasma Injections Decreases Pain and Improves Function in Patients with Symptomatic Knee Bone Marrow Lesions

**DOI:** 10.3390/biomedicines11071799

**Published:** 2023-06-23

**Authors:** Alan Ivković, Marin Glavčić, Filip Vuletić, Saša Janković

**Affiliations:** 1 Department of Orthopaedics and Trauma Surgery, University Hospital “Sveti Duh”, 10000 Zagreb, Croatia; 2 School of Medicine, University of Zagreb, 10000 Zagreb, Croatia; 3 Department of Clinical Medicine, University of Applied Health Sciences, 10000 Zagreb, Croatia; 4 Department of Orthopaedic and Trauma Surgery, University Hospital “Dubrava”, 10000 Zagreb, Croatia; 5 Faculty of Kinesiology, University of Zagreb, 10000 Zagreb, Croatia

**Keywords:** knee osteoarthritis, bone marrow lesion, arthroscopy, core decompression, autologous conditioned plasma, platelet rich plasma

## Abstract

The purpose of this prospective case series was to determine the effectiveness of using a combination of the core decompression and injection of autologous conditioned plasma (ACP) for the treatment of symptomatic knee bone marrow lesions (BML), as well as to report on the preliminary clinical results based on magnetic resonance imaging (MRI) and patient-reported outcomes (PROMs). Patients with OA-related BML who failed to improve on conservative treatment for three months underwent an identical procedure consisting of arthroscopy, core decompression, and the intraosseous injection of ACP and were followed up for 12 months. A statistically significant reduction in pain and an improvement in function, as measured by the Numeric Pain Rating Scale (NPRS) and Knee Injury and Osteoarthritis Outcome Score (KOOS), was observed at one-week follow-up (8.3 ± 0.8 to 1.5 ± 1.0; *p* ≤ 0.001 and 33.4 ± 10.6 to 53.9 ± 13.6; *p* ≤ 0.001 respectively). After six weeks, weight-bearing was allowed, but the trend did not change—the NPRS continued to be low (average 1.4 on 12-month follow-up) and the total KOOS increased 44.6 points from the baseline (average 78.0 on 12-month follow-up). The Whole-Organ Magnetic Resonance Imaging Score improved from 66.1 ± 19.4 prior to surgery to 58.0 ± 15.9 (*p* < 0.001) after 3 months. In our study, there was no control group, randomisation was not performed, and the sample size was relatively small. A combination of core decompression and the intraosseous injection of ACP into the affected subchondral area proved to be a safe and effective procedure that provides rapid pain relief and a significant increase in joint function up to one year postoperatively.

## 1. Introduction

Bone marrow lesions (BMLs) are changes in subchondral bone marrow signal intensity on magnetic resonance imaging (MRI). These changes are mainly characterised by a high signal on fluid-sensitive sequences such as T2-weighed, fat-suppressed, and short tau inversion recovery sequences. However, a low signal can occasionally be detected on T1-weighed images [1]. Historically, bone marrow oedema (BME) or bone marrow oedema syndrome (BMES) were used to describe excessive water signals in the marrow. However, data suggest that not only true oedema, but also a variety of other histopathological entities such as trabecular necrosis, cyst, fibrosis, and increased vascularisation can be present; hence, the more appropriate term BML is now used [2,3]. Indeed, numerous conditions are associated with the development of BMLs, including trauma, infections, and neoplasia. However, in many instances, BML is more elusive regarding its aetiology, course of disease, symptoms, and treatment options.

One of the most common varieties is the osteoarthritis (OA)-related BML, characterised by the alteration of subchondral bone remodelling [4]. Increased localised stress accompanied by the diminished regenerative capacity of subchondral bone results in biological and architectural changes typical for BML [5]. The hallmarks of clinical presentation are significant pain and rapid joint deterioration [6]. In addition to poor quality of life due to pain and disability, it has been postulated that patients with OA in the presence of BMLs have a much higher chance of progression to the need for knee arthroplasty [7]. It has been suggested that the early and targeted treatment of BMLs can alleviate pain and postpone the need for arthroplasty. Although various conservative and surgical options are available, there are no clear or standardised treatment guidelines. Core decompression has been traditionally used to treat BMLs. However, more advanced modalities, including adding calcium phosphate cement (CPC) injections or orthobiologics to core decompression, also provide promising results [8].

The use of ACP in the treatment of focal lesions in subchondral bone represents a promising new approach, although it has yet to be tested in terms of its long-term effectiveness through appropriate clinical trials. Our research allows us to see the potential benefit of using ACP in the treatment of BML and opens up the possibility of setting new hypotheses for further research.

The present study aimed to assess the efficacy of core decompression combined with the intraosseous injection of autologous conditioned plasma (ACP) and arthroscopy to achieve pain relief and improve the quality of life in patients with OA-related BMLs.

Hypothesis: core decompression combined with the intraosseous injection of ACP will achieve statistically significant pain relief and improve quality of life in patients with OA-related BMLs.

## 2. Materials and Methods

### 2.1. Patients and Study Design

The local ethical committee reviewed and approved the study, and all patients provided signed informed consent before the treatment. The study population consisted of 20 consecutive patients who failed to improve after three months of conservative treatment and met all of the inclusion criteria. There were no drop-offs during the study. The conservative protocol in this study is a slight modification of the one used by Simon et al. [9]. It consists of three consecutive monthly doses of 150 mg of ibandronate given orally (original protocol uses 3 mg of ibandronate intravenously), 1000 IU of vitamin D daily for three months, and partial weight-bearing during the first six weeks. Pain control was achieved with 1 g of paracetamol as needed. Potential candidates for the study were given an additional appointment for detailed screening by the senior authors to check the inclusion and exclusion criteria (see Table 1). The OA stage was determined using the Kellgren–Lawrence grading system (K-L) [10]. Patient demographics, Knee Injury and Osteoarthritis Outcome Score (KOOS), Numeric Pain Rating Scale (NPRS), and magnetic resonance imaging (MRI) were collected prospectively from the patient cohort from February 2018 to December 2020. In addition, lower extremity axis, meniscus status, and prior surgical treatment were noted. The location of the symptomatic BML was determined and recorded. All BMLs were assessed by semiquantitative, multi-feature Whole-Organ Magnetic Resonance Imaging Score (WORMS) [11]. The images were scored according to 14 independent articular features, but only the overall score and the subarticular bone marrow (SBM) abnormality are presented in the paper.

### 2.2. ACP Preparation

Arthrex ACP^®^ (Arthrex, Inc., Naples, FL, USA) specimens were collected according to the previously described protocol [12]. Briefly, a volume of 15 mL of whole venous blood was drawn into an Arthrex ACP^®^ Double-Syringe for a single spin in a centrifuge at 1500 rpm for 5 min. The volume of available ACP varied from 5 to 7 mL, and in all cases, the total volume of 5 mL of ACP was subsequently used for direct application into BML. ACP is considered a leukocyte-poor platelet-rich plasma (LP-PRP) with concentrations of platelets around 2–3× that of the whole blood.

### 2.3. Surgical Treatment

**Figure 1:**
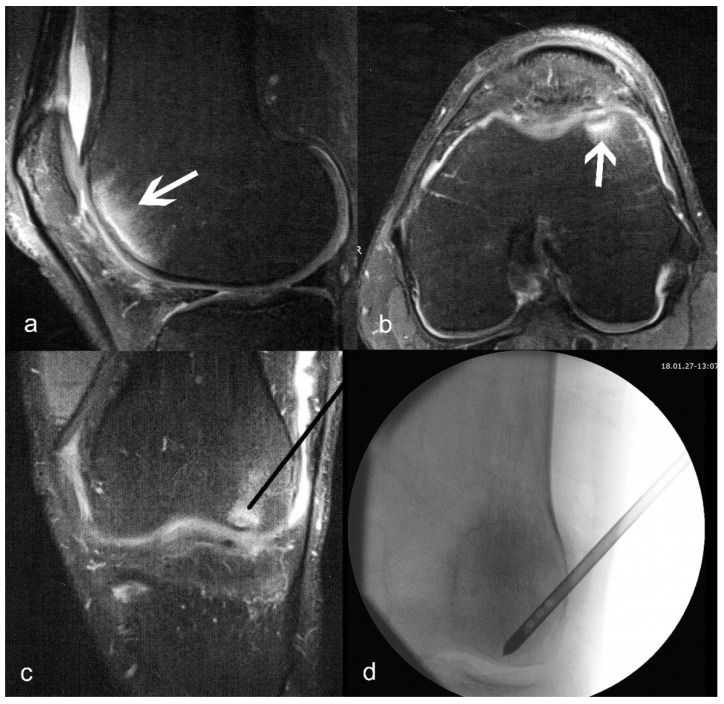
(**a**) Sagittal and (**b**) axial T2-weighted images of bone marrow lesion (shown with arrows) of the medial condyle; (**c**) planned position of the delivery canula placement (black line); (**d**) actual fluoroscopy indicating correct placement of the delivery canula.

The procedure was performed on a radiolucent table with a patient in the supine position. A thigh tourniquet was applied. The operative knee was positioned to facilitate arthroscopy and intra-operative radiographic image acquisition (Figure 1). Standard anteromedial (AM) and anterolateral (AL) portals were created, and a thorough examination of the knee joint was performed. Unstable cartilage or meniscal fragments were debrided with a shaver (Excalibur, Arthrex, Naples, FL, USA), but no other restorative procedures (e.g., microfracture) were performed at the time. For femoral lesions, a femoral anterior cruciate ligament (ACL) marking hook with a 2.4 mm guiding sleeve (RetroConstruction ACL Guide, Arthrex, Naples, FL, USA) was used to triangulate the approximate location of the lesion. Fluoroscopy was used throughout the procedure to confirm the position. A 2.4 mm guide pin (Arthrex, Naples, FL, USA) was advanced, and care was taken not to penetrate the subchondral bone and enter the joint space. The femoral marking hook was removed, and a 4.5 mm low-profile cannulated reamer (Arthrex, Naples, FL, USA) was advanced to the tip of the guide wire to complete core decompression. The reamer was removed, taking care not to remove the guide pin. An open-tip delivery needle (Arthrex, Inc., Naples, FL, USA) was placed over the guide pin (for closed-tip canula, the pin was removed, and the canula was inserted manually into the tunnel). The pin was removed, and 5 mL of ACP was injected into the lesion while slowly withdrawing the canula. Care needs to be taken not to apply too much pressure while injecting the ACP to avoid the fluid leaking out of the canal. For tibial lesions, an identical procedure was used, but without the femoral marking hook, and a closed-tip delivery needle is recommended.

### 2.4. Postoperative Protocol

Following the procedure, the patients were instructed to bear only 10% of the weight with crutch assistance for the next six weeks and then to increase weight-bearing by 10% each day during the next ten days. Physical therapy was initiated 10 to 14 days after the surgery, and a return to complete activities was allowed eight weeks postoperatively. All patients were advised to take 1000 international units (IU) of vitamin D3 per day and 1 g of paracetamol if needed for pain control.

### 2.5. Data Collection and Outcome Measures

For each patient, the baseline measures for NPRS, KOOS (total and five subscores: pain, symptoms, sports, activities of daily life/ADL, and quality of life/QoL), and WORMS were obtained before the procedure. The total KOOS score was calculated as the mean of all subscores. To assess the knee functionality and pain of the patient after the procedure, KOOS and NPRS were collected from each patient 1 week, 3 weeks, 6 weeks, 3 months, 6 months, and 12 months postoperatively. MRI and WORMS were repeated three months after the procedure. According to previous studies, we set the minimally clinical important difference (MCID) for NPRS as 2.0 [13]. Similarly, the MCIDs of KOOS-total, pain, symptoms, ADL, sports/recreation, and QoL were set as 10.0, 13.4, 15.5, 15.4, 19.6, and 21.1, respectively, according to a previous study [14].

### 2.6. Statistical Analysis

The analyses per time point were conducted using repeated-measures ANOVA with LSD test for pairwise comparisons between time points. In cases of violation of Mauchly’s sphericity assumption, a Greenhouse–Geisser correction was applied. The analysis for the WORMS variables was conducted using paired samples t-test, since these variables were only measured at two time points. All analyses were performed using R Statistical Software (v4.0.3; R Core Team 2020). [15] with rstatix 0.6.0. package [16] and Excel [17].

## 3. Results

### 3.1. Cohort Characteristics

The sample consisted of 20 consecutive patients who were treated with core decompression combined with the intraosseous injection of autologous conditioned plasma (ACP) and were monitored for 12 months after the surgery. In one patient, knee pain and swelling were observed at one-week follow-up, and no other complications were noted in the remaining patients. As summarised in Table 2, the average age of the patients was 60.9 ± 8.2 years, 45% of the patients were male, the average height was 172.3 ± 9.6 cm, the average weight was 88.9 ± 15.0 kg, and the average body mass index (BMI) was 30.0 ± 4.4.

### 3.2. Pain Improvement after the Procedure

**Figure 2:**
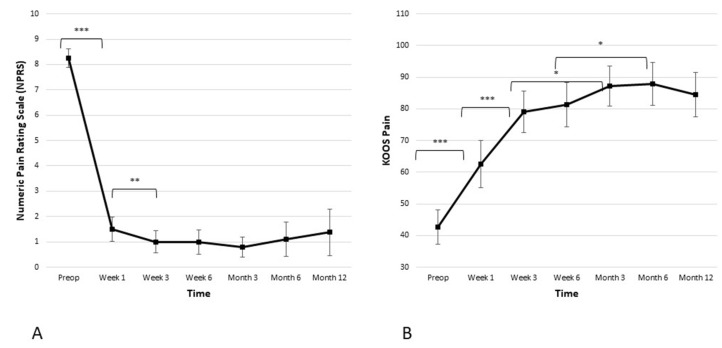
Significant reduction in pain is observed in both the (**A**) Numeric Pain Rating Scale (NPRS) and the (**B**) Knee Injury and Osteoarthritis Outcome Score (KOOS) pain subscale. The reduction is the most significant within the first 6 weeks when it reaches plateau and stays stable during the observed period of 12 months (* *p* < 0.05, ** *p* < 0.01, *** *p* < 0.001).

The average baseline NPRS score for the cohort was 8.3 ± 0.8. Figure 2 illustrates a statistically significant decrease in the pain score one week after the procedure (from 8.3 ± 0.8 preoperatively to 1.5 ± 1.0 in the week one; *p* ≤ 0.001). Another statistically significant decrease in the pain score was observed between week one and week three (from 1.5 ± 1.0 to 1.0 ± 0.9; *p* ≤ 0.05). After six weeks, weight-bearing resumed, but the curve stayed well below two points at the 3-, 6-, and 12-month time points. In addition, clinically meaningful improvement was observed in the KOOS pain subscore. There was a statistically significant improvement of the KOOS pain subscore in weeks one and three (from 42.6 ± 11.6 preoperatively to 62.6 ± 15.9 in week one, and then to 79.0 ± 14.0 in week three; *p* ≤ 0.001 and *p* ≤ 0.001 respectively). The KOOS pain subscore reached a plateau at the 3-month time point and remained stable over the 6- and 12-month time points postoperatively.

### 3.3. Improvement of KOOS Scores (Function, ADL, QoL, Sport) after the Procedure

**Figure 3:**
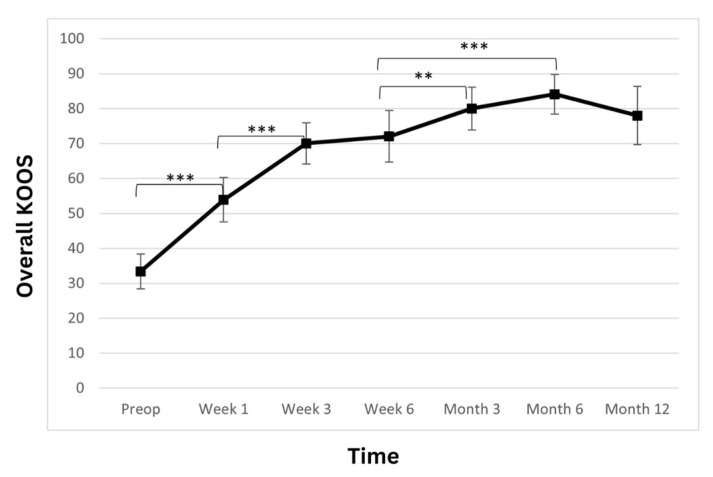
Overall Knee Injury and Osteoarthritis Outcome Score (KOOS) significantly improved. The improvement is linear during the first 3 weeks, and the improvement continues until the 6-month time point, when a slight reduction is observed. However, the score remains high at the 12-month time point (** *p* < 0.01, *** *p* < 0.001).

The total KOOS score showed a statistically significant improvement over the observed period (Table 3; Figure 3). At admission, the average patient score was 33.4 ± 10.6. At weeks one and three, a postoperatively statistically significant increase in the scores was observed (53.9 ± 13.6; *p* < 0.001 and 70 ± 12.6; *p* < 0.001, respectively). At week six, the score further improved to 72.0 ± 15.7 (*p* > 0.05). Another statistically significant improvement was observed at the 3-month follow-up, where the score rose to 80 ± 13.1 (*p* < 0.01) and then remained stable at 6- and 12-month time points (84.1 ± 12.2 *p* > 0.05 and 78 ± 17.9 *p* > 0.05 respectively).

**Figure 4:**
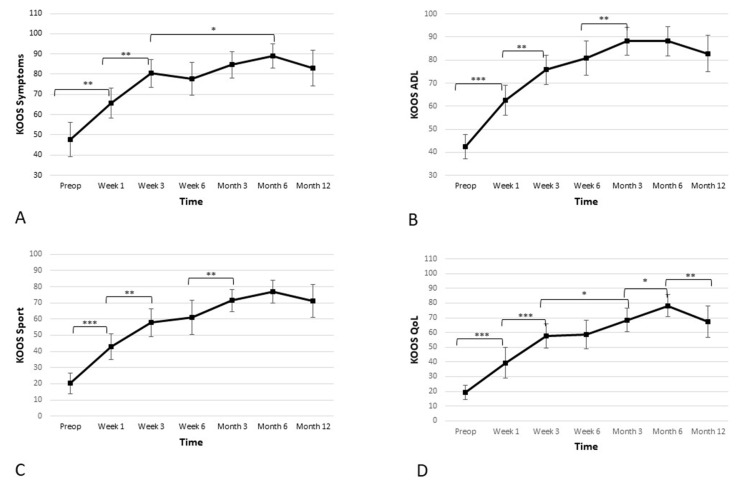
Significant improvement in the four additional subscales—(**A**) symptoms, (**B**) activities of daily living (ADL), (**C**) sport, and (**D**) quality of life (QoL)—of the Knee Injury and Osteoarthritis Outcome Score (KOOS) is observed. The improvement is linear during first 3 weeks, and the improvement continues until the 6-month time point, when a slight reduction is observed. However, the score remains high at the 12-month time point (* *p* < 0.05, ** *p* < 0.01, *** *p* < 0.001).

Apart from the pain subscore, the KOOS scale consists of four additional subscores, which were assessed separately. Those four additional subscores were: activities of daily living (ADL), quality of life (QoL), sports and recreation, and symptoms. Further results and statistics regarding KOOS subscores can be found in Table 3 and Figure 4.

### 3.4. Improvement of WORMS MRI Scores after the Procedure

**Figure 5:**
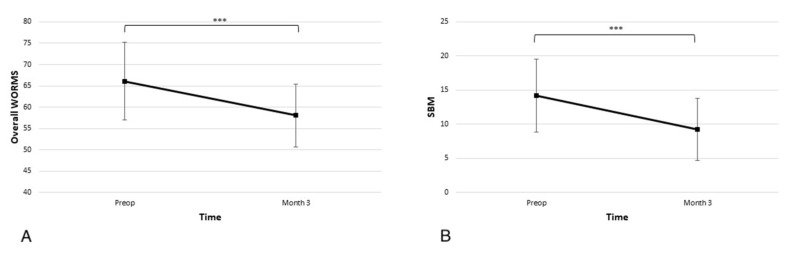
Significant improvements in the (**A**) overall Whole-Organ Magnetic Resonance Imaging Score (WORMS) and (**B**) subarticular bone marrow (SBM) abnormalities subscore are observed at the 3-month time point (*** *p* < 0.001).

In the overall WORMS scale, the average score at admission was 66.1 ± 19.4, and the subscore for SBM abnormality was 14.2 ± 11.4. As seen in Figure 5 at the 3-month time point, there was a statistically significant improvement in the overall score and SBM subscore (58.0 ± 15.9 *p* < 0.001 and 9.3 ± 9.7 *p* < 0.001).

## 4. Discussion

The main finding of our study was that patients with knee OA-related BML treated with a combination of core decompression and injection of ACP demonstrated rapid and significant improvements in pain and function, which persisted over the next 12 months. These overall results indicate that it is a feasible biologic option for the initial treatment of patients with BML and a potential alternative for postponing a total knee replacement (TKR).

It has been shown that OA patients with BMLs have a higher risk of rapid advancement toward end-stage disease requiring arthroplasty. In a prospective study by Tanamas et al. [4], the authors included 132 patients who were followed for four years. They concluded that patients with more severe BMLs had more significant cartilage loss after the two years and an increased risk of advancement toward TKR. Even though TKR is a reliable procedure with significant pain relief and improvement in function and quality of life, it is a major procedure associated with significant healthcare costs, complications, and prolonged rehabilitation [18]. Hence, it is essential to consider less invasive, biologic options for treating BMLs associated with degenerative changes.

A very high level of pain is the hallmark symptom of BMLs, and it does not correspond with normal pain levels in patients with knee OA without BML. From the work of Uchio et al. [19], we know that the intraosseous pressure in patients with osteonecrosis is twice as high as in those with medial compartment knee OA. In combination with the slower drainage of venous blood and an abundance of nociceptors in the subchondral bone region, it is understandable that high pain levels are such a remarkable feature of this clinical condition. The understanding of this cascade led to the introduction of core decompression for the treatment of BMLs, first in hip and then in knee patients as well [20]. The good outcomes might be the result of the improvement in venous drainage, reduction in pressure, and easier inflow of nutrients due to the more favourable arteriovenous pressure difference. At the same time, having access to the BML region via a special delivery canula (see Figure 1) at the time of core decompression enables the surgeon to add biologic cues to the decompressed area to facilitate the recovery of the affected bone.

Platelet-rich plasma (PRP) can be used as an addition to the core decompression to facilitate the recovery of the affected bone. The main advantages of this biologic approach are that it is entirely autologous, has no immune response, and is cost efficient. Adding growth factors and chemokines to the core decompression provides the BML region with the requisite signals for tissue recovery and regeneration [21].

Sanchez et al. [22] introduced the technique in 2016 with a pilot study where they compared two treatment modalities: three intraarticular injections of PRP versus one intraosseous injection of PRP followed by two intraarticular injections of PRP one week apart. They used LP-PRP preparations with moderate platelet concentrations (2–3 times above baseline), no leukocytes, and no erythrocytes. They reported an improvement in the overall KOOS score and a significant reduction in pain the subscore as well in VAS. The same group of authors conducted an observational study with two treatment groups: three intraarticular infiltrations of PRP every week versus a combination of two intraosseous PRP infiltrations, with the first intraarticular injection followed by two more intraarticular injections in the following two weeks after the intraosseous infiltrations [23]. Again, they used LP-PRP preparations with moderate platelet concentrations (1.5–2.5 times above baseline). They concluded that the combination of intraarticular and intraosseous injection was not clinically superior (measured by KOOS and WOMAC) at 2 months, but demonstrated superiority at 6 and 12 months.

In a three-way randomised control trial (RCT), Su et al. tested the hypothesis that the addition of the intraosseous injection of PRP to traditional intraarticular treatment with PRP or hyaluronic acid (HA) would add a positive effect. They used a leukocyte-rich-platelet-rich plasma (LR-PRP) preparation method with a mean platelet concentration 5.61-fold greater than that of the whole blood. The first group of patients received two intraosseous injections of 2 mL of LR-PRP and two intraarticular injections of LR-PRP 14 days apart. The second group received two intraarticular injections of 6 mL of LR-PRP 14 days apart. The third group received an intraarticular injection of 2 mL of commercially available HA (five administrations, each one week apart). They concluded that the combination of intraosseous and intraarticular injections resulted in significantly superior clinical outcomes within 18 months of follow-up.

The most recent addition to the ongoing development of the method is a prospective cohort study performed by Lychagin et al. [24]. The study involved 17 patients treated with an intraosseous application of 5 mL of LP-PRP. A significant decrease in pain scores and an increase in functional PROMs (WOMAC and KOOS) were reported during the 12-month follow-up. An interesting addition to the study was an evaluation of the changes in serum cartilage oligomeric matrix protein (COMP), which is usually used as an informative marker of cartilage metabolism.

**Figure 6:**
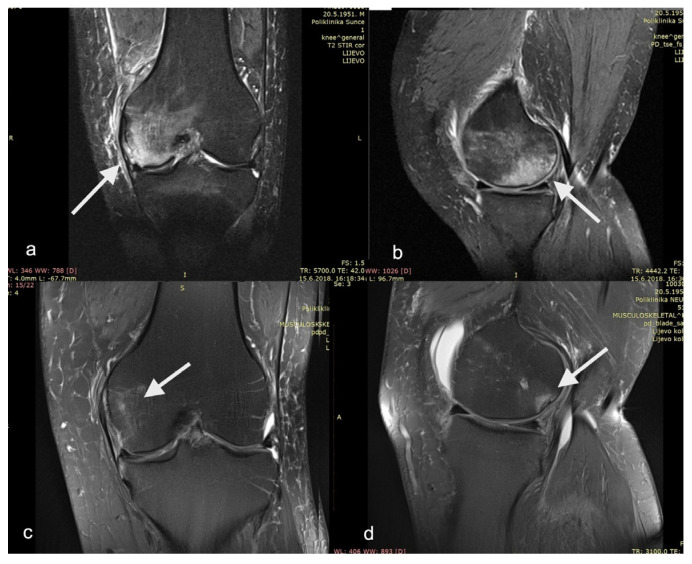
T2−weighted magnetic resonance imaging of patient’s left knee as (**a**) AP view and (**b**) LL view before surgery showing medial femoral condyle bone marrow lesion. The same patient in (**c**) AP view and (**d**) LL view at 12−month follow−up. Bone marrow lesions are show with arrows.

Although all of the mentioned studies are rather heterogeneous in terms of the study design, PRP preparation method used, and PROMs measured, they all resulted in a significant decrease in pain and increase in functional scores. Indeed, our study has comparable outcomes in that respect. Within a few weeks, the patients in this study reported a statistically significant decrease in pain and statistically significant improvement in all functional subscores. We believe this rapid improvement is initially due to the synergistic effect of core decompression (lowering the intraosseous pressure) and non-weight-bearing (decrease in mechanical overload). The sustained positive effect, which reaches a plateau around six months postoperatively, might be attributed to the action of instilled PRP (anabolic and anti-catabolic effect of growth factors and cytokines). The slight reduction in the functional parameters between month 6 and month 12 might be due to the effective return to the activities of daily life and repetitive biomechanical overload from being overweight. The twelve-month follow-up T2-weighted knee MRI of one of our patients shows an improvement in the medial femoral condyle BML (Figure 6).

Similar to other studies, our study observed a high prevalence of overweight patients in the cohort. The BMI was 30.0 ± 4.4, and only 2 out of 20 patients (10%) were in the normal range for age and gender. This implies significant biomechanical overload, which contributes to the development of BMLs and the deterioration of knee OA.

MRI is crucial for adequately diagnosing OA-related BMLs, and it should be considered part of the routine diagnostic protocol in those OA patients with unusually high pain levels. It is best detected on T2-weighted (T2w), or proton density-weighted (PDw), fat-suppressed (FS) fast spin-echo (FSE), or short tau inversion recovery (STIR) sequences and is characterised by areas of high signal intensity [10]. The WORMS score is based on a semiquantitative, multi-feature scoring method that is applicable to conventional MRI techniques. Although it is rather complex and incorporates 14 features, our study was able to focus on the subarticular bone marrow (SBM) abnormality score as being central to the pathophysiology of BML. MRI was performed three months after the procedure, and the results indicate significant overall improvement and improvement in the SBM subscore.

Another interesting biologic option is to use bone marrow concentrate (BMC) as an addition to core decompression. BMC is an autologous product rich in progenitors and has been successfully used as the intraosseous delivery option in bone fracture healing and the treatment of femoral head avascular necrosis [25]. Hernigou et al. [26] designed a prospective RCT that was carried out on 30 patients and 60 knees presenting with bilateral OA secondary to knee osteonecrosis (ON) related to the use of steroids. During the same anaesthesia, one knee received TKR, and the other received a subchondral injection of BMC. The average follow-up was 12 years, and the authors concluded that, while both knees had similar functional outcomes, BMC-treated knees resulted in a lower incidence of complications and quicker recovery. In another prospective case series, Vad et al. [27] treated ten knee OA patients with an injection of BMC into the chondral–bone interface of the affected compartment. They concluded that the subgroup of patients with a combination of BML and OA had the best outcomes.

Finally, in an attempt to address the structural integrity of subchondral bone, which is the leading site of the pathological process, a subchondroplasty (SCP) procedure has been introduced [28]. SCP is performed under fluoroscopic guidance and consists of injecting calcium phosphate cement into the BML. The goal of the procedure is to provide a mechanical environment for bone remodelling and regeneration. In a consecutive patient series (*n* = 66) treated with SCP, Cohen and Sharkey [29] reported durable improvement in pain and functional scores over two years. In addition, 70% of patients at two years’ follow-up did not elect to undergo TKR. In another study, Bonadio et al. [30] reported a significant improvement in patient-reported outcomes (VAS and KOOS) for a short follow-up of 6 months, and Chua et al. [31], in a more recent study, reported significant improvements in WOMAC, KOOS, and VAS scores following SCP. However, Chatterjee et al. [32] reported unfavourable outcomes in “a concerning proportion” of their patients, and they advised against SCP for patients with advanced OA changes.

This study has important limitations. There was no control group, randomisation was not performed, and the sample size was relatively small. Although we made an effort to apply exactly 5 mL of obtained ACP, no additional analysis to determine the exact number of applied cells and concentration of growth factors was performed. Furthermore, NPRS and KOOS are based on patient-reported, inherently subjective data. To offset this limitation, we performed MRIs, but only at one time point. The long-term observation of this cohort is still ongoing, and we hope to provide better insight into the true potential of the proposed method in the years to follow.

## 5. Conclusions

MRI is mandatory for the diagnosis and development of an adequate treatment strategy for BML patients. This study showed that core decompression along with the injection of ACP into the affected subchondral area after only one week provides rapid pain relief and statistically significant improvement in joint function and quality of life, as assessed by NPRS, KOOS, and WORMS (*p* < 0.001, *p* < 0.001, and *p* < 0.001, respectively). The beneficial effect of the procedure was the most remarkable during the first three months and the measured outcomes remained stable for the length of the study. The procedure is simple, reproducible, and safe, with only one minor complication observed during the study.

## Figures and Tables

**Table 1:** Inclusion and exclusion criteria. (NSAID, nonsteroidal anti-inflammatory drugs; HA, hyaluronic acid; BML, bone marrow lesion; OA, osteoarthritis).

Inclusion Criteria	Exclusion Criteria
Knee pain for at least 2 months	Active immunosuppressive and anticoagulant therapy
Failure of conservative protocol for 3 months	Systemic autoimmune rheumatic diseases and blood disorders
Presence of hypercaptating lesion (>2 cm in diameter) in T2-weighted fat suppression images located in the subchondral region of the tibial or femoral condyle	Tricompartmental OA of the affected knee
VAS pain in the compartment of BML at least 4/10	Obvious structural deficiencies in the to be treated subchondral region
Moderate to severe OA (2 to 3 according to Kellgren–Lawrence) in the same compartment as the BML	Deviation of the mechanical alignment of the lower limbs larger than 8 degrees
Age 40–70 years	

**Table 2:** Demographic characteristics of patients with bone marrow lesions (BML). (BMI, body mass index).

Variable	*N* (%) or Mean ± SD
Age, y (38–74)	60.9 ± 8.2
Sex (male)	9 (45%)
Height, cm (158–186)	172.3 ± 9.6
Weight, kg (69–115)	88.9 ± 15.0
BMI, kg/m^2^(24.1–37.9)	30.0 ± 4.4

**Table 3:** Means and standard deviations across time points. (ADL, activities of daily life; QoL, quality of life; KOOS, Knee Injury and Osteoarthritis Outcome Score; NPRS, Numeric Pain Rating Scale; SBM, subchondral bone marrow).

Time	KOOS						NPRS	WORMS	
	ADL	Pain	QoL	Sport	Symptoms	Total		SBM	Total
Preop	42.4 ± 11.3	42.6 ± 11.6	19.4 ± 10.	20.2 ± 13.5	47.7 ± 18.2	33.4 ± 10.6	8.3 ± 0.8	14.2 ± 11.4	66.1 ± 19.4
Week 1	62.5 ± 14.0 ***	62.6 ± 15.9 ***	39.4 ± 22.0 ***	42.9 ± 16.8 ***	65.8 ± 15.9 **	53.9 ± 13.6 ***	1.5 ± 1.0 ***		
Week 3	75.8 ± 13.5 ***	79.0 ± 14.0 ***	57.8 ± 17.7 **	57.8 ± 18.6 **	80.4 ± 14.5 **	70.0 ± 12.6 ***	1.0 ± 0.9 *		
Week 6	80.8 ± 16.0	81.4 ± 15.0	58.4 ± 20.8 *	61.0 ± 22.2	77.7 ± 17.3	72.0 ± 15.7	1.0 ± 1.0		
Month 3	88.2 ± 13.1 **	87.3 ± 13.6	68.6 ± 17.3	71.6 ± 14.6 *	84.6 ± 13.8	80.0 ± 13.1 **	0.8 ± 0.8	9.3 ± 9.7 ***	58.0 ± 15.9 ***
Month 6	88.2 ± 13.7	87.8 ± 14.5	78.1 ± 16.1 *	76.9 ± 14.8	89.0 ± 12.7	84.1 ± 12.2	1.1 ± 1.4		
Month 12	82.8 ± 16.8	84.5 ± 15.0	67.6 ± 22.8 **	71.3 ± 21.6	83.0 ± 19.1	78.0 ± 17.9	1.4 ± 2.0		

* *p* < 0.05, ** *p* < 0.01, *** *p* < 0.001.

## Data Availability

The data that support the findings of this study are available on request from the corresponding author (M.G.). The data are not publicly available due to their containing information that could compromise the privacy of the research participants.
